# Stain susceptibility of a giomer and a particulate-filled injectable composite with one-step and multi-step polishing techniques, and color recovery after brushing and repolishing

**DOI:** 10.1186/s12903-026-09651-2

**Published:** 2026-08-25

**Authors:** Eman Samy Ibrahim, Reham Mohamed Salem, Khaled Aly Nour

**Affiliations:** Department of Operative Dentistry, Faculty of Dentistry, Ain Shams University, Cairo, Egypt

**Keywords:** Giomer, Injectable composite, ΔE_00_, Polishing systems, Stain susceptibility, Color recovery

## Abstract

**Objectives:**

This study evaluated the stain susceptibility and color recovery of Giomer and highly filled injectable composite compared with a nano-filled packable composite, using one-step and multi-step polishing systems.

**Methods:**

Ninety composite specimens were prepared from Filtek Z350, Beautifil Flow Plus, and G-ænial Injectable and divided into three polishing subgroups (*n* = 10): Mylar strip, OneGloss, and Enamel Plus Shiny. After baseline color measurement (T_0_), the specimens were immersed in coffee at 37 °C for 30 days. Color changes were measured using a Vita EasyshadeV spectrophotometer after brushing using a soft toothbrush (ΔE_brushing; T₁–T₀) and after repolishing using AL_2_0_3_ paste (ΔE_repolishing; T₂–T₀). Data were analyzed using a linear mixed model with Yeo–Johnson transformation and Holm’s correction (α = 0.05).

**Results:**

A significant interaction was observed among material type, polishing system, and color change (*p* = 0.020). G-ænial Injectable showed the lowest stain susceptibility (ΔE_brushing = 2.84; ΔE_repolishing = 1.88). Multi-step polishing significantly improved stain resistance for all tested materials. Repolishing significantly reduced discoloration in all groups (*p* < 0.001) by increasing L* values and reducing shifts in a* and b* coordinates. Among all materials, only the multi-step-polished G-ænial Injectable achieved color change values that approached the acceptability threshold (ΔE₀₀ ≤ 1.8).

**Conclusion:**

Highly filled injectable composites and multi-step polishing improved stain resistance and color recovery of resin composites, whereas S-PRG fillers and matrix-set surfaces increased stain susceptibility.

**Clinical relevance:**

Multi-step polishing enhances esthetics, while repolishing improves restoration longevity.

**Supplementary Information:**

The online version contains supplementary material available at 10.1186/s12903-026-09651-2.

## Introduction

Long-term esthetic performance remains a concern, as color change and surface degradation are leading causes of restoration replacement in daily practice. Increasing patient expectations for natural and durable esthetics have made color stability a critical determinant of restorative success and longevity [1, 2].

The discoloration of resin-based restorative materials is influenced by both intrinsic and extrinsic factors. Intrinsic factors are primarily related to material composition, including resin matrix hydrophilicity, degree of conversion, filler loading, filler particle size, and the integrity of the filler–matrix interface [3]. Extrinsic factors include dietary chromogens, oral hygiene habits, and surface roughness [4, 5]. Discoloration generally occurs through stain adsorption or absorption, with stain uptake referring to surface stains adsorped on the material’s surface [6, 7]. In contrast, stain absorption involves the penetration of pigments into the resin matrix or organic phase of the material, making discoloration more resistant to routine cleaning and potentially requiring professional repolishing or restoration replacement [6, 8].

Among dietary staining agents, coffee is considered one of the most potent contributors to discoloration of resin composites because of its high chromogenicity, low polarity, and ability to penetrate the polymer network, resulting in both surface and subsurface staining [6, 7]. Consequently, coffee immersion is widely used in laboratory studies to simulate long-term staining challenges. The color change observed after the staining procedure is typically evaluated using instrumental methods like spectrophotometry. These methods are reported to provide more accurate and objective assessments than visual evaluation [9].

Advances in nanotechnology have led to the continuous development of restorative materials with enhanced mechanical and esthetic properties [10]. Nanofilled composites, such as Filtek Z350 XT, were developed to combine superior polishability with improved mechanical performance by incorporating nanosized fillers and nanoclusters. Despite their favorable aesthetics, their TEGDMA-containing matrix and the potential degradation of nanocluster fillers may increase their susceptibility to water and staining over time [5, 11].

Giomer restorative materials incorporate surface pre-reacted glass-ionomer (S-PRG) fillers, providing bioactive properties such as fluoride release and recharge. However, the inclusion of S-PRG technology may also increase water sorption and alter surface integrity, potentially compromising long-term color stability and polish retention [11]. In contrast, highly filled injectable composites such as G-ænial Universal Injectable were introduced, offering improved handling and filler-resin balance [12, 13]. Their homogeneously dispersed ultrafine silane-treated fillers have been reported to improve gloss retention and provide a self-polishing effect over time [14]. Nevertheless, evidence regarding their resistance to staining and color recovery after stain removal procedures remains limited.

These three categories of restorative materials differ substantially in filler structure, filler loading, resin matrix composition, polishability, and water sorption behavior, all of which may influence stain susceptibility and long-term clinical esthetics [15]. Although several studies have independently evaluated nanofilled composites, giomers, or injectable composites, comparative evidence assessing their behavior under identical staining and polishing conditions remains scarce. Furthermore, limited data are available on how differences in filler architecture and surface properties affect stain-removal efficiency (color recovery) after brushing or repolishing.

In addition to material composition, finishing and polishing protocols significantly impact stain retention. Stain retention refers to stains that remain adsorbed to the surface and resist removal by mechanical cleaning methods; it doesn’t involve the deliberate reduction of the material’s surface. It reflects surface reactivity, integrity, and polishability, indicating long-term color stability and clinical maintenance [16]. Recent evidence suggests that the abrasive particle composition of polishing systems significantly influences the color stability (ΔE_00_) and surface integrity of resin composites. Aluminum oxide (Al₂O₃)-based systems and diamond particle-based systems differ in hardness, cutting efficiency, and interaction with filler–matrix structures [17].

The OneGloss polishing system is a simplified one-step polishing system utilizing aluminum oxide abrasives. While one-step aluminum oxide systems offer clinical convenience and reduced chairside time, previous studies have reported that they may produce rougher surfaces and lower gloss values compared with multi-step or diamond-based polishing systems [18]. Conversely, diamond abrasive particles possess greater hardness and cutting efficiency, enabling more uniform abrasion of both filler particles and the resin matrix, thereby producing smoother, more highly polished surfaces [9]. Meanwhile, there’s no consensus on whether simplified one-step systems can achieve aesthetic outcomes comparable to those of conventional multi-step systems, especially for restorative materials with different filler technologies [19, 20].

In clinical practice, restorations frequently undergo surface degradation and staining over time, making stain removal strategies clinically relevant [21]. There are several methods for removing superficial stains from resin composites, including brushing at home, professionally by repolishing, and bleaching. However, toothbrushing depends primarily on the type of dentifrice used and the pressure applied by the patient. Consequently, toothbrushing is a slower process for removing discolorations. Bleaching and repolishing can quickly remove discoloration from teeth, professionally and independently of the patient [3]. The efficacy of such interventions is clinically significant, as it influences the perceptibility (ΔE_00_ > 0.8) and acceptability (ΔE_00_ > 1.8) thresholds that dictate restoration longevity and patient satisfaction [22, 23].

Despite the growing interest in esthetic maintenance of resin-based restorations, there remains a lack of comparative evidence evaluating the stain susceptibility and color recovery of Giomer, nanofilled, and highly filled injectable resin composites in relation to their distinct filler structures and polishability characteristics. Additionally, few studies have compared the effectiveness of one-step aluminum oxide polishing systems and diamond-based polishing systems, particularly polishing pastes, in restoring the color stability of these materials after staining.

Therefore, this study investigated the effect of restorative material composition and polishing technique on the stain susceptibility of resin composite materials. In addition, the effectiveness of regular toothbrushing and professional repolishing in restoring the color of stained restorations after exposure to common staining solutions was evaluated. By clarifying how different filler technologies interact with various polishing systems, this study aims to provide clinically relevant guidance to improve restoration longevity and minimize unnecessary replacement. The null hypothesis was that neither material type nor polishing protocol would significantly affect stain susceptibility and color recovery of the resin composite.

## Materials and methods

### Sample size calculation

The sample size was determined using G*Power 3.1.9.4. A minimum of 10 specimens was necessary to identify a statistically significant difference at the 5% significance level. The effect size was calculated as 0.4, with α set at 0.05, and power (β) at 0.85. Consequently, the total required sample size was *N* = 90, with *n* = 10 for each subgroup.

### Sample grouping

A total of 90 composite specimens were prepared and divided into 9 groups (*n* = 10) according to two study levels. Level 1: Resin composite materials; 3 groups: *nano-filled Filtek Supreme Z350 XT Universal Restorative*, *nano-hybrid Beautifil Flow Plus X F00*, and *G-ænial Universal Injectable composite*. (Table 1) provides a summary of the resin composite materials used in this study, including their composition and type.

**Table 1:** Materials, compositions, manufacturer, and LOT number. Details from the manufacturer’s data [5, 23]

Material name, type and shade	Filler	Monomer	Manufacturer and LOT no.
Filtek Supreme Z350 XT Universal Restorative Nano-filled packable universal composite. Shade: A2 body	Non-agglomerated/non-aggregated 20 nm silica filler non-agglomerated/non-aggregated 4–11 nm zirconia filler Aggregated zirconia/silica cluster filler (comprised of 20 nm silica and 4 to 11 nm zirconia particles). The Enamel shades average cluster particle size of 0.6–10 μm **Filler content**: 78.5 Wt %, 63.3 vol%.	Bis-GMA, UDMA, TEGDMA, bis-EMA (resins, and PEGDMA).	3 M ESPE, St. Paul, Minnesota, USA. **Lot no.**: NF24629
Beautifil Flow Plus X F00 Nano-Hybrid, Injectable Composite with GIOMER Technology. Shade: A2	Multifunctional glass filler and S-PRG filler based on fluroroaluminosilicate glass. Particle size range: 0.01-4.0 Mean particle size: 0.8 μm. **Filler content**: 67.3wt%, 47.0 vol%	Bis-GMA (15wt%), TEGDMA (13wt%).	Shofu Inc., Kyoto, Japan. **Lot no.**: 022371, 022370
G-ænial Universal Injectable composite (nano-hybrid) Highly filled flowable composite. Shade: A2	Silica, strontium and barium glass. Particle size range: 10–200 nm **Filler content**: 69 wt%-81wt%, 50% vol	**Matrix**: UDMA, bis-EMA (Bis-MPEPP), methacrylate monomers, UV-light absorber **Others**: Pigments, photo initiator.	GC Corp., Tokyo, Japan. **lot no.**: 2,212,221

*Bis-GMA* Bisphenol A Glycidyl Methacrylate, *UDMA* Urethane dimethacrylate, *EMA* Ethyl methacrylate, *TEGDMA* Triethylene glycol dimethacrylate, *PEGDMA* polyethylene glycol dimethacrylate, *Bis-MPEPP* bisphenol A polyethylene glycol polypropane diol dimethacrylate, *S-PRG* surface pre-reacted glass, *wt%* weight%, *vol%* volume percent, *µm* micrometer, *Nm* nanometer, *UV* Ultraviolet

**Fig. 1:**
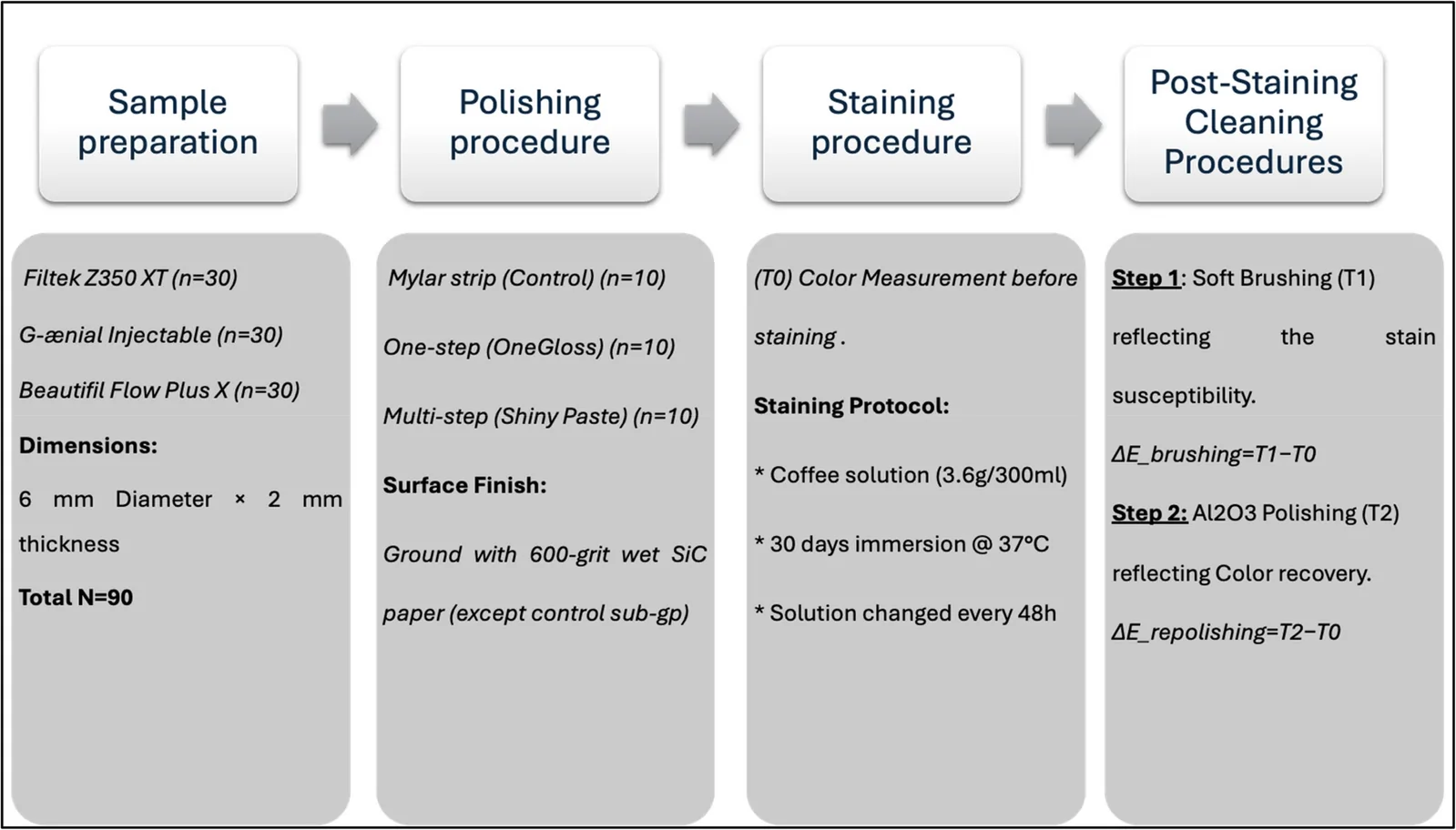
Diagram for the sequential steps of the methodology

**Fig. 2:**
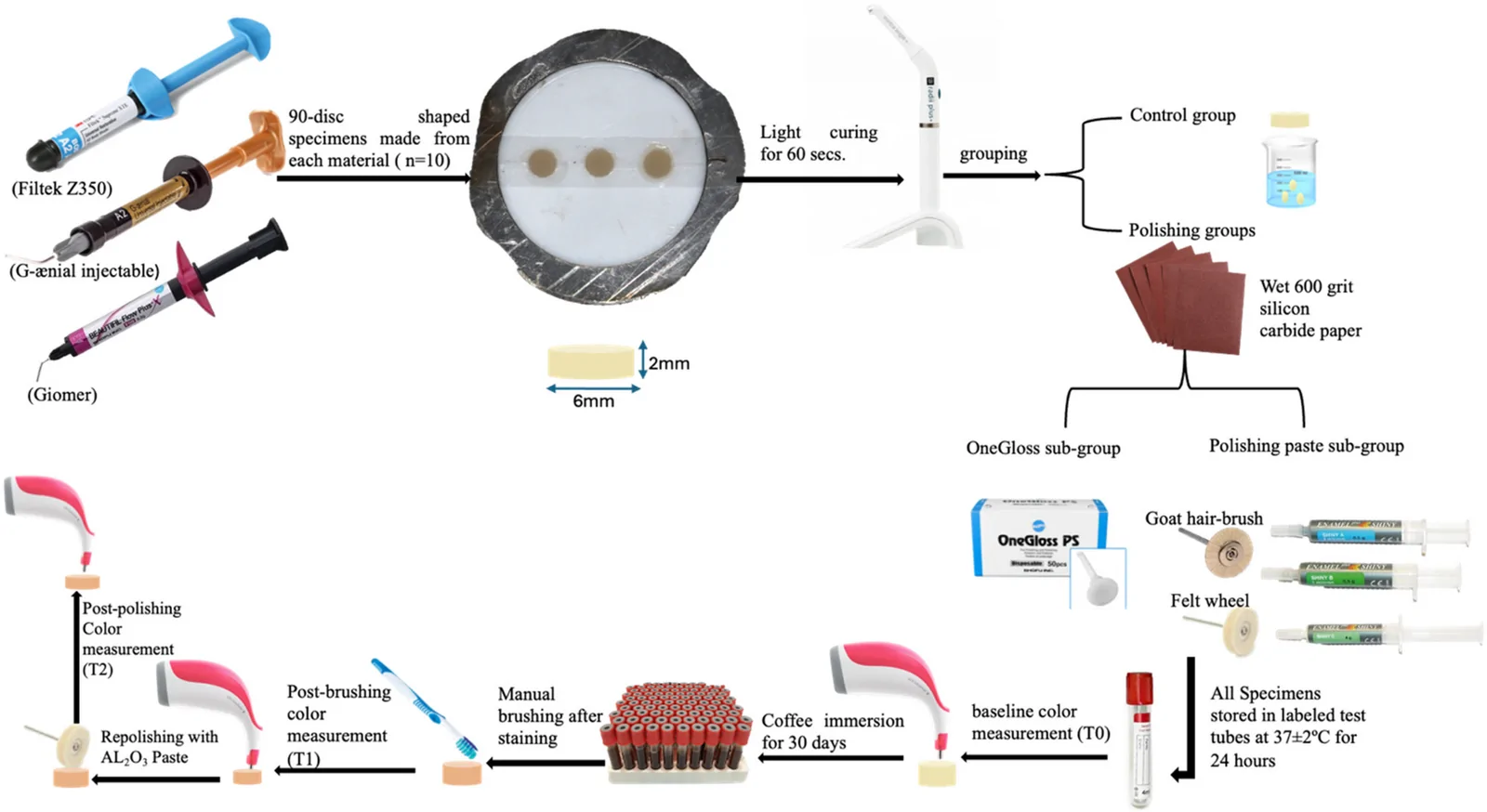
Schematic representation of the study design

Level 2: Polishing protocol; 3 subgroups: Mylar strip (*Rite-dent*,*Mfg. Corp*,*USA*) (Control group), One step (*OneGloss*^®^*polishing discs*), and Multi-step (*Enamel Plus Shiny diamond polishing pastes*). (Table 2) provides a summary of the polishing protocols used in this study, including their composition and Direction of usage per manufacturer instructions. (Fig. 1) illustrates the sequential steps of the methodology, while (Fig. 2) provides a visual overview of the study design. 

**Table 2:** Composition, list, and direction of usage of the polishing systems. Details from the manufacturer’s data [24, 25]

Polishing system	OneGloss^®^ (Inverted cone disc)	Enamel Plus Shiny (pastes)
No of the application steps	One-step	Three-step
Matrix	Synthetic rubber (Polyvinyl siloxane)	Polyethylene glycol
Abrasives	Aluminum oxide (Al_2_O_3_)and Silicone oxide (SiO_2_)grains	**Shiny A**: Diamond powder applied with a goat hair brush Shiny S **Shiny B**: Diamond powder applied with a goat hair brush Shiny S **Shiny C**: Aluminium oxide (Al_2_O_3_) powder applied with the disc felt Shiny F
Particle size	Mean alumina particle size 85 μm	Shiny A: 3 μm Shiny B: 1 μm Shiny C: < 1 μm
Direction of usage	pressure equivalent to 0.3 N, speed between 3,000 rpm to 10,000 rpm with intermittent water spray,	pastes should be applied sequentially from coarser to finer paste, Under dry conditions for 20 s.
Manufacturer	Shofu, Japan	Micerium, Italy.

### Specimen preparation

A split Teflon mold was used to obtain composite discs with a diameter of 6 mm and a thickness of 2 mm. A glass slide was placed with adhesive tape on top to fix the mold in position during specimen fabrication. A mylar strip (Rite-dent, Mfg. Corp., USA) about 60 microns thick was adjusted before placing the Teflon mold on it [26, 27] (Fig. 2). The packable composite (Filtek Z350 XT) was placed into the mold holes and adapted with a double flat to obtain a uniform surface with no irregularities and to remove any excess. In contrast, the injectable composites (Beautifil Flow Plus X F00 and G-ænial Universal Injectable) were injected incrementally through the mold holes, from the bottom upward to facilitate complete mold filling and ensure the absence of voids or irregularities. All specimens were subsequently covered with another mylar strip, gently pressed with another glass slide for 1 min to flatten the surface and remove any excess before curing.

The composite was polymerized for 20 s according to the manufacturer’s instructions using the Radii Plus LED Curing Light (SDI, Bayswater, Victoria, Australia) with a curing tip diameter of 10 mm and an intensity of 1400 mW/cm^2^, with a Mylar strip and glass slide in place. The curing tip was in direct contact with the glass slide and perpendicular to it. Following this, additional polymerization was conducted on the specimens for 20 s after removing the strip and glass slide. The output intensity was checked every 5 specimens using a radiometer to ensure it was ≥ 1400 mW/cm^2^ [28]. The mold was then disassembled, each sample was inspected for visible voids after removal, and the bottom of the disc was labeled. Any excess material on the edges of the polymerized discs was carefully trimmed without altering the Mylar-formed surface.

### Finishing and polishing procedures

Each group was randomly subdivided into 3 sub-groups with 10 composite discs each. A control group with no further treatment and 2 experimental sub-groups. Composite discs were fixed in place on adhesive tape over a glass slide for better control, where finishing and polishing procedures would be performed.

The surface of the experimental sub-group’s composite disc, cured against the mylar strip, was manually ground for 30 s using wet 600-grit silicon carbide paper in a circular motion. It was then rinsed with an air-water spray for 10 s and air-dried for 5 s, representing the finishing step before polishing [29]. To minimize variation, the same operator performed all polishing procedures using the same low-speed handpiece (Strong Dental Co., Ltd, Taiwan) at 3000 rpm. A digital kitchen scale (Model: SF-400, China) was used as a pressure guide, with a light intermittent pressure of 30–40 gm (approximately 0.3 newtons) indicating the required pressure for the procedure. Each stroke was made in the same direction, and polishing instruments were discarded after each use. Application of each polishing sub-group (OneGloss discs & Enamel plus shiny polishing pastes) was performed following the manufacturer’s instructions. All Specimens were rinsed with an air-water spray for 10 s, air-dried for 5 s, incubated in labeled test tubes at 37 ± 2 °C for 24 h, measured for baseline color, and then immersed in the coffee staining solution [29] (Fig. 2).

### Baseline color measurements (T0)

All composite samples were measured for baseline color using a Vita EasyshadeV spectrophotometer (Vita Zahnfabrik, Bad Sackingen, Germany) that implements the Commission Internationale de l’Éclairage L*a*b* system [30]. The device was fully charged and calibrated per the manufacturer’s recommendations before each measurement. Under the same lighting conditions, the spectrophotometer tip was placed perpendicular to the center of each sample, and the initial color values were measured against a black background. Each sample’s shade measurement was repeated 4 times and calibrated after each measurement; the average value was then recorded [23, 31].

### Staining procedures

3.6 g of coffee (Nescafé Classic, Nestle, Switzerland) were dissolved in 300 milliliters of boiling water, mixed for ten minutes, and then filtered through a solution filter paper to create the coffee solution. Prior to immersing the specimens, the prepared solution was tempered in an incubator for approximately 30 min to reach 37 ± 2 °C (body temperature). Each specimen was individually immersed in the coffee solution and maintained at a constant temperature (37 ± 2 °C) throughout the 30-day staining period to simulate the average intraoral temperature under standardized laboratory conditions. The coffee solution was replaced every 48 h [9, 32]. Samples were immersed in the coffee solution for 30 days, following previous studies, as an accelerated staining protocol to produce sufficient intraoral discoloration [33, 34].

### Post-staining cleaning procedures and color measurement

Color was remeasured after two sequential cleaning steps: Step 1 – Soft Brushing (T1) & Step 2 – Al₂O₃ Polishing (T2). Following the final repolishing procedure, all specimens were thoroughly rinsed with an air-water spray for 10 s to remove residual polishing debris, then air-dried for 5 s before the final color measurement.

(Table 3) Provide a concise overview of the cleaning procedures and their descriptions.

**Table 3:** Post-staining cleaning procedures steps and description

Stage	Procedure	Instrument	Description	Defined ΔE Variable
Step 1 – Soft Brushing (T1)	Gentle brushing without toothpaste	Soft toothbrush under controlled load in one direction in a circular motion for 30 s.	Simulates light mechanical removal of weakly adsorped surface stains. (cleaning at home)	**ΔE_brushing =** reflection for stain susceptibility. **ΔE_brushing**= After 1-month staining (T₁–T₀).
Step 2 – Al₂O₃ Polishing (T2)	Polishing using aluminum oxide paste (Shiny C)	Felt wheel at 10,000–12,000 rpm for 20 s.	Simulates professional surface cleaning removing firmly attached stains.	**ΔE_repolishing =** reflection for color recovery. **ΔE_repolishing** = After repolishing (T₂–T₀).

Mean L* a* b* values were calculated and compared across three measurement time points (baseline, after brushing, and after repolishing, where L* (Lightness) value ranges from zero (Black) to 100 (white), a* ( Red-green axis), and b* (Blue-Yellow axis). Color change values post-staining (ΔE_00_) after brushing and repolishing of each specimen were calculated according to the CIEDE2000 equation using an online Delta E calculator [23, 35] (Figs. 1 and 2):

## Results

### Statistical analysis

Continuous data were presented as mean and standard deviation (SD). Normality was assessed by examining the distribution and using the Shapiro-Wilk test, which indicated normality. A linear mixed model was used to assess the effects of color changes. Residual normality was evaluated using the methods described earlier. Homoscedasticity was assessed by visual inspection of the fitted-versus-residuals plot, violating both assumptions. The data were transformed using the Yeo-Johnson transformation, improving the model fit and reducing substantial deviations from normality in the residuals, though mild heteroscedasticity remained. A new mixed model was constructed with a cluster-based robust covariance matrix to account for heteroscedasticity. All subsequent analyses were performed on transformed data, with the original scale reported in summary statistics for interpretability. Fixed effects were evaluated using Wald tests, and post hoc comparisons were conducted using Wald-type tests with Holm’s multiple comparisons adjustment. Effect sizes were quantified using the Robust Effect Size Index (RESI). The significance level was set at *p* < 0.05. R statistical analysis software version 4.5.2 for Windows was used for statistical analysis.

#### Effect of different variables and their interaction

A significant three-way interaction was found among the tested resin composite material, polishing system, and color change (*p* = 0.020), with a small effect size of 0.21. Material (*p* = 0.002), polishing system (*p* < 0.001), and color change (*p* < 0.001) showed significant main effects (Table 4).

**Table 4:** Effect of different variables and their interactions on color change (ΔE)

Source	df	χ^2^	*p*-value	RESI (95% CI)
Restorative material	2	12.20	0.002*	**0.24 (0.10 to 0.40)**
Polishing system	2	24.37	< 0.001*	**0.35 (0.21 to 0.51)**
Color change measurement	1	177.66	< 0.001*	**1.00 (0.85 to 1.15)**
Material * Polishing	4	3.77	**0.437ns**	**0.00 (0.00 to 0.25)**
Material * Measurement	2	12.14	0.002*	**0.24 (0.10 to 0.40)**
Polish * Measurement	2	0.36	**0.834ns**	**0.00 (0.00 to 0.15)**
Material * Polishing * Measurement	4	11.62	0.020*	**0.21 (0.03 to 0.38)**

*df* degree of freedom, *RESI* Robust Effect Size Index, *CI* Confidence interval, *ns *not significant

* significant* (p<0.05)*

#### Effect of restorative material on the color change (ΔE_00_) after brushing and repolishing

**Fig. 3:**
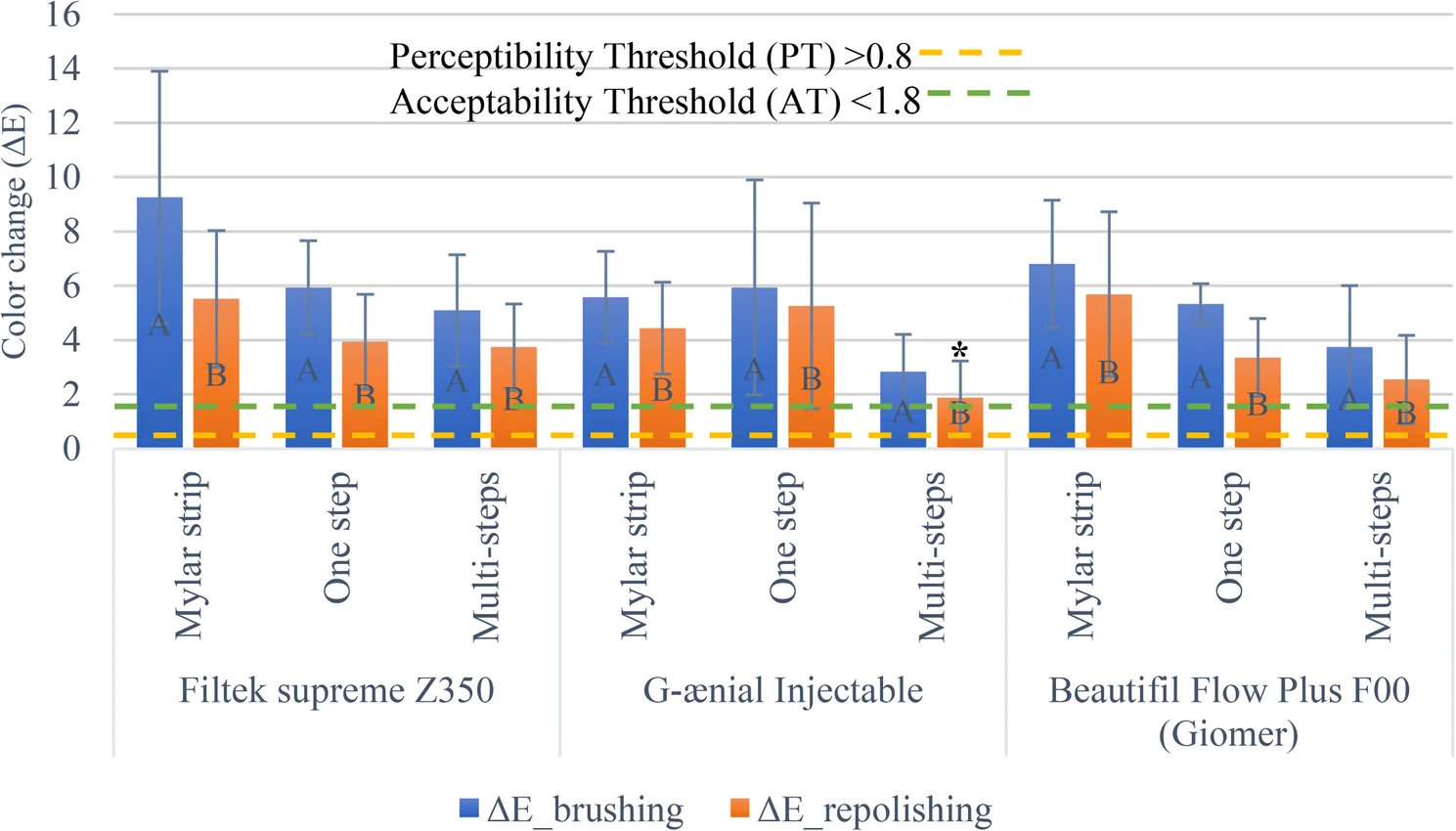
Bar chart showing mean and standard deviation values of Color change (ΔE00), perceptibility threshold (PT), and acceptability threshold (AT) after brushing and repolishing for different resin composite materials across polishing systems. Measurements with different superscripts within the same material and polishing system differ significantly, * showing the material is within an acceptable threshold

Post hoc pairwise comparisons using Wald-type tests showed that Filtek Supreme Z350 XT had significantly higher color change (ΔE_00_) values than G-ænial Injectable. Material differences were significant in multi-step groups after both brushing and repolishing (*p* < 0.05), with medium effect sizes (RESI = 0.27 & 0.31), respectively. After soft brushing (ΔE_brushing), Filtek Z350 XT was the most significant, exhibiting the highest color change of (5.09 ± 2.06), followed by Beautifil Flow Plus X F00 with color change of (3.74 ± 2.26), while G-ænial Injectable had the lowest color change of (2.84 ± 1.36). After repolishing (ΔE_repolishing), Filtek Z350 remained the highest (3.74 ± 1.59), while G-ænial Injectable retained the lowest values (1.88 ± 1.35) (Table 5; Fig. 3).

**Table 5:** Comparisons and summary statistics of color change (ΔE_00_) for different types of resin composite under different polishing protocols

Measurement	Polishing	Color change (ΔE_00_) (Mean ± SD)			p-value	RESI (95% CI)
		Filtek supreme Z350	G-ænial Injectable	Beautifil Flow Plus X F00 (Giomer)		
ΔE_brushing	Mylar strip	9.25 ± 4.65^A^	5.57 ± 1.69^A^	6.80 ± 2.35^A^	**0.058ns**	**0.20 (0.00 to 0.44)**
	One step	5.93 ± 1.72^A^	5.94 ± 3.96^A^	5.33 ± 0.75^A^	**0.680ns**	**0.00 (0.00 to 0.26)**
	Multi-steps	5.09 ± 2.06^A^	2.84 ± 1.36^B^	3.74 ± 2.26^AB^	0.014*	**0.27 (0.06 to 0.50)**
ΔE_repolishing	Mylar strip	5.52 ± 2.52^A^	4.43 ± 1.69^A^	5.69 ± 3.03^A^	**0.494ns**	**0.00 (0.00 to 0.30)**
	One step	3.94 ± 1.75^A^	5.25 ± 3.79^A^	3.36 ± 1.43^A^	**0.511ns**	**0.00 (0.00 to 0.30)**
	Multi-steps	3.74 ± 1.59^A^	1.88 ± 1.35^B^	2.55 ± 1.63^AB^	0.005*	**0.31 (0.11 to 0.54)**

Values with different superscripts within the same horizontal row are significantly different

*RESI* Robust Effect Size Index, *CI* Confidence interval, *ns* not significant

* significant (*p*<0.05)

#### Effect of polishing system on the color change (ΔE_00_) of tested restorative materials after brushing and repolishing

Post hoc pairwise comparisons using Wald-type tests showed that the multi-step polishing protocol consistently produced significantly lower ΔE_00_ values for all resin composite materials after both brushing and repolishing (*p* < 0.05), with a large effect size for the G-ænial Injectable group (0.44). For Filtek Supreme Z350 XT, there was a significant difference among polishing systems after brushing, with the Mylar strip showing the most significant color change, followed by OneGloss, and Multi-step showed the least values (*p* = 0.036), with a small effect size (0.23). However, there was no significant difference between different polishing systems after repolishing (*p* = 0.119), and the effect size was small (0.16). For G-ænial Injectable, there was a significant difference among polishing systems after both brushing and repolishing, with OneGloss and Mylar strip showing nearly equal color change values, while the Multi-step polished group had the lowest values (*p* < 0.001), with a large effect size (RESI > 0.41). For Beautifil Flow Plus X F00, there was a significant difference among polishing systems after both brushing and repolishing with a medium effect size, where the Mylar strip showed the most significant color change values, while the Multi-step group showed the least values (*p* = 0.006, RESI = 0.31) & (*p* = 0.015, RESI = 0.27), respectively (Table 6; Fig. 3)**.**

**Table 6:** Comparisons and summary statistics of color change (ΔE00) for different polishing systems

Measurement	Material	Color change (ΔE_00_) (Mean ± SD)			*p*-value	RESI (95% CI)
		Mylar strip	One step	Multi-steps		
ΔE_brushing	Filtek supreme Z350	9.25 ± 4.65^A^	5.93 ± 1.72^AB^	5.09 ± 2.06^B^	0.036*	**0.23 (0.00 to 0.47)**
	G-ænial Injectable	5.57 ± 1.69^A^	5.94 ± 3.96^AB^	2.84 ± 1.36^B^	< 0.001*	**0.44 (0.24 to 0.66)**
	Beautifil Flow Plus X F00 (Giomer)	6.80 ± 2.35^A^	5.33 ± 0.75^A^	3.74 ± 2.26^B^	0.006*	**0.31 (0.10 to 0.53)**
ΔE_repolishing	Filtek supreme Z350	5.52 ± 2.52^A^	3.94 ± 1.75^A^	3.74 ± 1.59^A^	**0.119ns**	**0.16 (0.00 to 0.41)**
	G-ænial Injectable	4.43 ± 1.69^A^	5.25 ± 3.79^A^	1.88 ± 1.35^B^	< 0.001*	**0.41 (0.21 to 0.63)**
	Beautifil Flow Plus X F00 (Giomer)	5.69 ± 3.03^A^	3.36 ± 1.43^AB^	2.55 ± 1.63^B^	0.015*	**0.27 (0.06 to 0.50)**

Values with different superscripts within the same horizontal row are significantly different

*RESI* Robust Effect Size Index, *CI* Confidence interval, *ns* not significant

* significant (*p*<0.05)

#### Effect of brushing and repolishing post-staining on the color change (ΔE_00_) of tested restorative materials

All tested materials showed significantly higher ΔE_00_ values after brushing than after repolishing (*p* < 0.001), Effect sizes were large for most comparisons (RESI > 0.45). ΔE_00_ values decreased significantly across the multi-step polished groups after repolishing. While the ΔE_repolishing of Filtek Z350 XT (3.74 ± 1.59) and ΔE_repolishing of Beautifil Flow Plus X F00 (2.55 ± 1.63) still exceeded the clinically accepted threshold, G-ænial Injectable (1.88 ± 1.35) fell within the clinically acceptable threshold (Table 7; Fig. 3).

**Table 7:** Comparisons and summary statistics of color change (ΔE_00_) difference after brushing with color change after repolishing

Material	Polishing	Color change (ΔE_00_) (Mean ± SD)		*p*-value	RESI (95% CI)
		ΔE_brushing	ΔE_repolishing		
Filtek supreme Z350	Mylar strip	9.25 ± 4.65^A^	5.52 ± 2.52^B^	< 0.001*	**0.59 (0.39 to 0.80)**
	One step	5.93 ± 1.72 ^A^	3.94 ± 1.75 ^B^	< 0.001*	**0.80 (0.60 to 1.02)**
	Multi-steps	5.09 ± 2.06 ^A^	3.74 ± 1.59 ^B^	0.002*	**0.31 (0.12 to 0.54)**
G-ænial Injectable	Mylar strip	5.57 ± 1.69 ^A^	4.43 ± 1.69 ^B^	< 0.001*	**0.39 (0.19 to 0.61)**
	One step	5.94 ± 3.96 ^A^	5.25 ± 3.79 ^B^	0.005*	**0.28 (0.09 to 0.50)**
	Multi-steps	2.84 ± 1.36 ^A^	1.88 ± 1.35 ^B^	< 0.001*	**0.49 (0.30 to 0.71)**
Beautifil Flow Plus F00 (Giomer)	Mylar strip	6.80 ± 2.35 ^A^	5.69 ± 3.03 ^B^	0.001*	**0.32 (0.13 to 0.55)**
	One step	5.33 ± 0.75 ^A^	3.36 ± 1.43 ^B^	< 0.001*	**0.45 (0.26 to 0.67)**
	Multi-steps	3.74 ± 2.26 ^A^	2.55 ± 1.63 ^B^	< 0.001*	**0.55 (0.35 to 0.77)**

Values with different superscripts within the same horizontal row are significantly different

*RESI* Robust Effect Size Index, *CI* Confidence interval

* significant (*p*<0.05)

#### Color-coordinate data (L*, a*, b*) for tested restorative materials across polishing systems

**Fig. 4:**
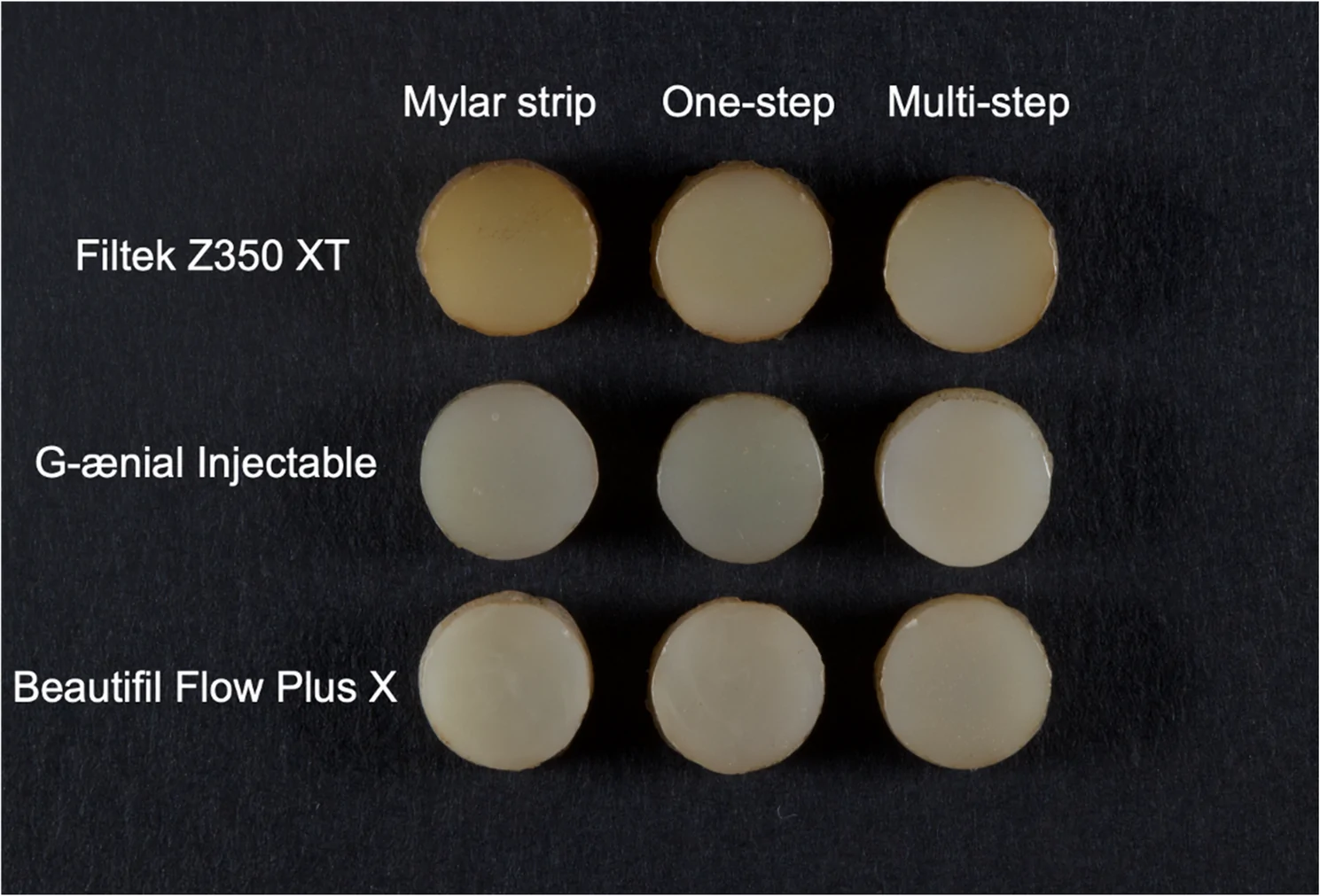
Perceptible color changes of the tested restorative materials across polishing systems after 30 days of immersion and repolishing procedure

**Fig. 5:**
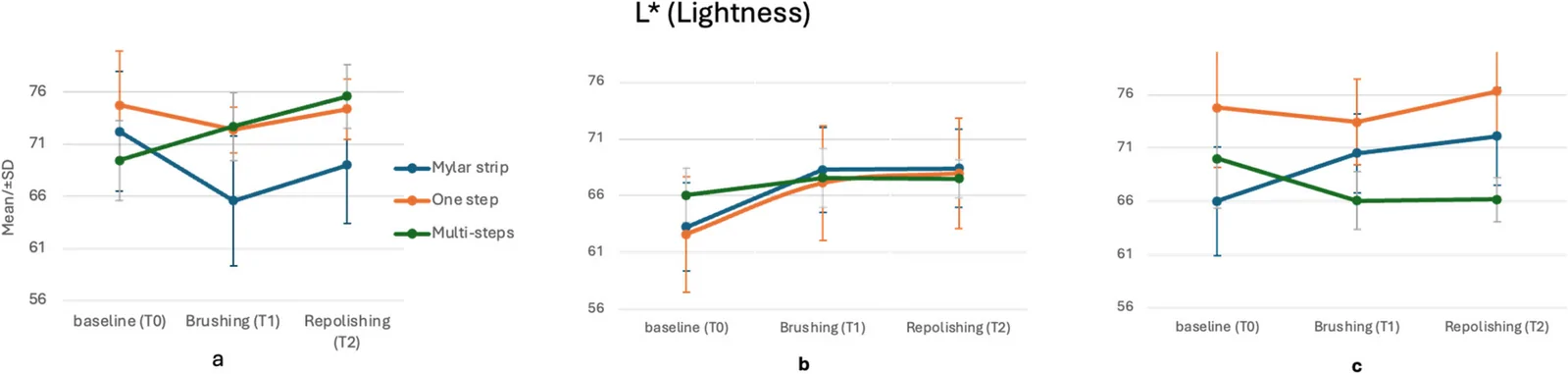
Line chart showing mean and standard deviation values for the L* (Lightness) coordinate of different materials across polishing systems at the three measurement time points. (**a**) L* (Lightness) coordinate changes in Filtek Z350 XT, (**b**) L* (Lightness) changes in G-ænial Injectable, and (**c**) L* (Lightness) changes in Beautifil Flow Plus X (Giomer)

**Fig. 6:**
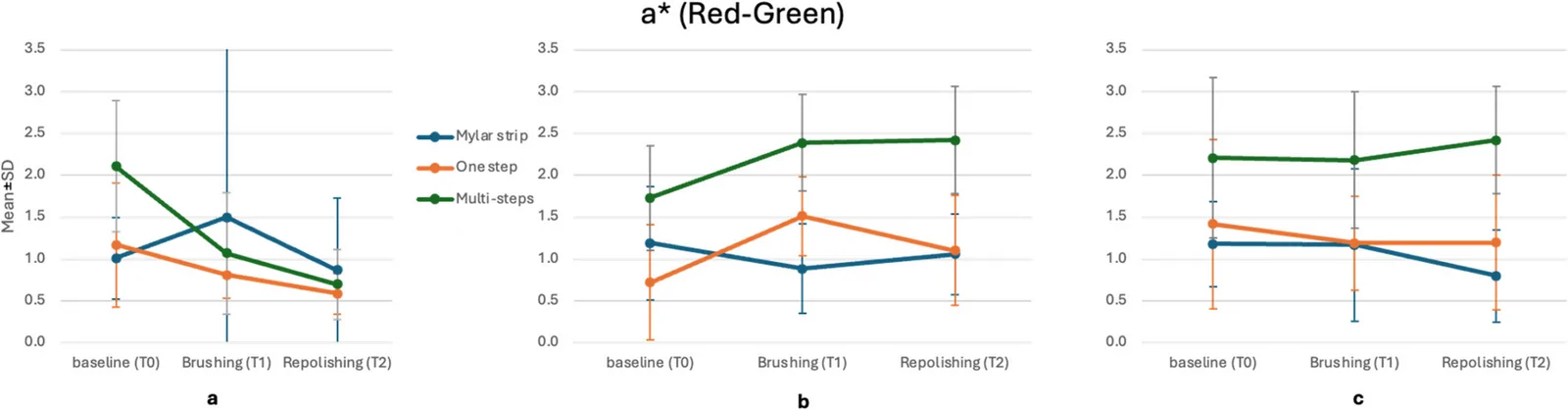
Line chart showing mean and standard deviation values for the a* (Red-Green) coordinate of different restorative materials across polishing systems at the three measurement time points. (**a**) a* (Red-Green) coordinate changes in Filtek Z350 XT, (**b**) a* (Red-Green) changes in G-ænial Injectable, and (**c**) a* (Red-Green) changes in Beautifil Flow Plus X (Giomer)

**Fig. 7:**
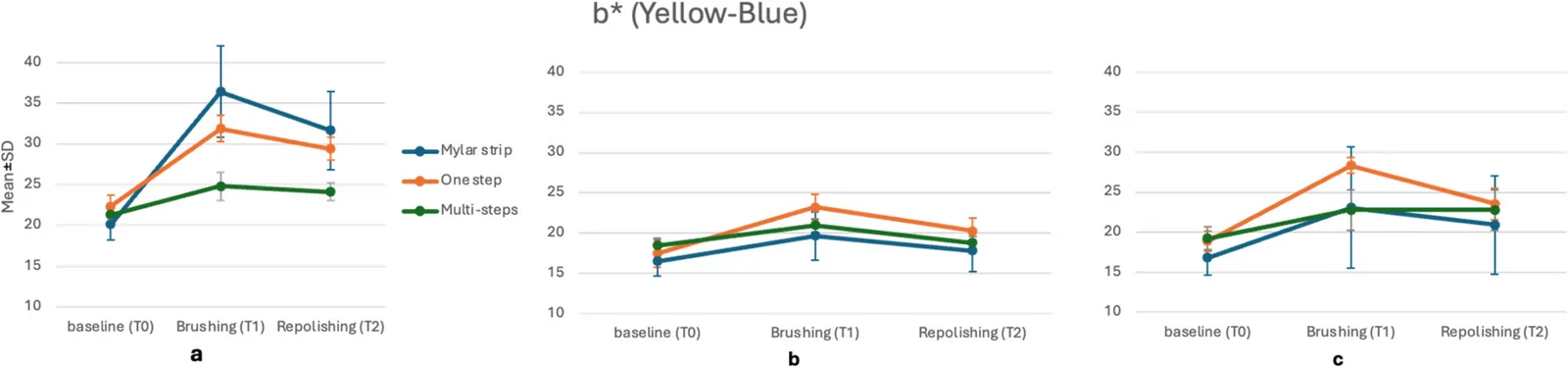
Line chart showing mean and standard deviation values for the b* (Yellow-blue) coordinate of different restorative materials across polishing systems at the three measurement time points. (**a**) b* (Yellow-blue) coordinate changes in Filtek Z350 XT, (**b**) b* (Yellow-blue) changes in G-ænial Injectable, and (**c**) b* (Yellow-blue) changes in Beautifil Flow Plus X (Giomer)

Analysis of the CIE L*, a*, and b* coordinates revealed material- and polishing-dependent color changes after brushing and repolishing. Brushing generally reduced L* values and altered a* and b* coordinates, indicating darker, more chromatically shifted surfaces. Filtek Supreme Z350 XT exhibited the greatest changes in coordinates, whereas G-ænial Injectable showed the least. Multi-step polishing produced the smallest deviations in color coordinates across all materials. Repolishing partially restored the original optical characteristics by increasing L* values and reducing shifts in a* and b* coordinates, consistent with the significant reduction in ΔE_00_ values observed after repolishing (*p* < 0.001) (Figs. 4, 5, 6 and 7).

## Discussion

The present study evaluated the stain susceptibility and color recovery of two different resin composites: nano-hybrid Beautifil Flow Plus X F00 (Giomer) and highly filled flowable G-ænial Universal Injectable, compared to nano-filled Filtek Supreme Z350 XT, after using two distinct polishing systems. Our findings revealed that the material composition, polishing protocol, and post-staining cleaning procedure (brushing versus repolishing) significantly influenced the color change (ΔE_00_). Notably, a significant three-way interaction (*p* = 0.020) confirmed that the degree of discoloration was influenced by both the intrinsic material properties and the surface treatment. Therefore, the null hypothesis was rejected.

Coffee was chosen as the staining solution for a 30-day immersion period due to its potent chromogens and yellow pigments, which have a lower molecular weight than those in tea or cola. As suggested by Ertas et al., 24 h of coffee immersion is equivalent to one month of coffee consumption. Therefore, the 30-day immersion period approximates 2.5 years of clinical exposure and provides sufficient time for substantial stain accumulation [33]. This duration was meticulously designed to maximize specimen staining, enabling strong adsorption of surface stains that can’t be removed by brushing or repolishing. Specimens were incubated at body temperature (37 ± 2 °C) to simulate oral conditions as closely as possible while maintaining standardized experimental conditions throughout the study. This approach eliminated temperature as a potential confounding factor and minimized the coffee’s pronounced chromogenic effect [23, 34]. Solutions were replaced every 48 h to prevent bacterial growth, maintain staining potency, and simulate repeated exposure to fresh staining agents [9, 32, 34].

The post-staining cleaning procedure was performed first using a soft toothbrush in circular motions for 30 s without toothpaste to avoid altering the surface, simulating regular oral hygiene at home. The extent to which brushing reduces extrinsic pigmentation or increases roughness depends on several factors, including the characteristics of the resin matrix and filler particles, the duration of brushing, and the abrasiveness of the toothpaste [36]. Therefore, we excluded toothpaste as a potential factor influencing the outcome. It was assumed that brushing the surface after staining and before the second color measurement (T_1_) would remove the weakly adsorped surface stains without altering the surface itself [36]. In agreement with Cinelli et al., brushing would avoid inaccurate readings regarding the overall stain level and instead focus on the surface adsorped stains retained on the surface of the materials after the immersion period [5].

Repolishing was performed using AL_2_O_3_ paste (Shiny C) and a felt-wheel brush, as these are typically formulated with very fine particles, often less than a few microns [37]. These particles are suitable for composite polishing without causing excessive abrasion, thereby enabling color recovery. Anfe et al. reported that staining is confined to a superficial layer of less than 20 μm. Removing the first 20 μm significantly reduced ΔE_00_ values, and the color remained stable after further wear. This suggests that staining affects an area of less than 20 μm and that repolishing can restore the surface color [38]. To assess stain susceptibility and color recovery, color change values were used as indicators. The CIE Lab color system, using the CIEDE2000 formula (ΔE_00_), is considered a reliable indicator of human perceptibility and acceptability of color differences, especially for small color shifts. According to Paravina et al., the CIEDE2000 50:50% PT is set at 0.8, and the CIEDE2000 50:50% AT is set at 1.8 [9, 22, 31].

Regarding the effect of resin composite material on the stain susceptibility, only a significant difference between the three tested restorative materials was observed in the multi-step polished groups. The lowest statistically significant color change value (ΔE_00_) was found at G-ænial Injectable both after brushing and repolishing (ΔE_brushing = 2.84 ± 1.36) & (ΔE_repolishing = 1.88 ± 1.35), exhibiting relatively improved optical behavior, with L* values improved following repolishing procedures and minimal a* variation suggesting excellent chromatic stability in the red-green axis (Table 5; Fig. 3) (Figs. 5, 6 and 7). In contrast, Filtek Z350 XT exhibited the most significant color change after brushing (T_1_) (ΔE_brushing = 5.09 ± 2.06) and greater variability in L*a*b* coordinates among polishing protocols, suggesting increased yellow discoloration. Moreover, significant color change values persisted after repolishing (T_2_) (ΔE_repolishing = 3.74 ± 1.59). Although repolishing reduced discoloration, L*a*b* values remained elevated, suggesting deeper or more persistent stain penetration compared with the other materials (Fig. 4).

This outcome may be attributed to the chemistry and microstructure, particularly resin hydrophilicity, degree of conversion, and filler-matrix bond. This aligns with the findings of Nasim et al. [1] and Cinelli et al. [5], who reported that nanofilled composites are particularly stain-prone due to their Triethylene glycol dimethacrylate (TEGDMA) rich resin matrix and the incomplete silanization of nanocluster fillers, which facilitate water sorption and pigment penetration [39]. Lower staining susceptibility has been associated with reduced water sorption and improved post-polishing surface smoothness. It was reported that stain sorption exhibited a strong correlation with water sorption [40]. Moreover, stain retention and color recovery are closely related to material polishability and filler characteristics. This aligns with a study by Farghal et al., which investigated the stain susceptibility of similar resin composites [23]. The explanation for the reduced staining potential lies in the technology employed by G-ænial Injectable, which ensures the dense packing of smaller filler particles or enhanced silane bonding between fillers and the organic matrix [41]. Its persistence in Filtek Z350 after repolishing reflects degradation of nanoclusters that may occur during polishing, resulting in irregularities on the restoration surface [42]. These irregularities can affect the surface’s smoothness and promote the accumulation of surface stains [42, 43].

Furthermore, the improved optical behavior of G-ænial Injectable, with L* values improved following repolishing procedures and minimal a* variation, suggests excellent chromatic stability in the red-green axis. This is attributed to its reduced hydrophilicity due to the stain-resistant co-monomer urethane dimethacrylate (UDMA) and to its optimized filler-resin interface [26]. Although staining increased yellow chromaticity after brushing, repolishing reduced b* values, indicating effective removal of surface stain and predominantly extrinsic discoloration. G-ænial Injectable composites showed superior surface stain resistance and better color recovery compared to Giomer and nanofilled groups. Furthermore, brushing reduced color change values in all groups; all specimens remained above the clinically acceptable threshold (ΔE_00_ > 1.8), indicating that routine oral hygiene measures alone were insufficient to completely reverse discoloration after prolonged coffee exposure [6]. Repolishing improved color recovery by removing retained surface stains; however, only the multi-step polished G-ænial Injectable group approached clinically acceptable values (ΔE_repolishing = 1.88 ± 1.35). This suggests that while brushing may effectively reduce superficial extrinsic stains, professional repolishing is more effective in improving restoration esthetics, particularly for materials with superior polishability and lower water sorption [23] (Table 7; Fig. 3). In accordance with our research, previous studies by Jrady et al. and Meniawi et al. demonstrated that Filtek Universal composites exhibited the most significant color change, while injectable composites demonstrated superior stain resistance because they lack BIS-GMA in their components, with no filler debonding after acidic challenges [44, 45].

On the other hand, Beautifil Flow Plus X F00 (Giomer) color change values were highly significant than G-ænial Injectable while significantly lower compared to Filtek Z350 XT (Table 5; Fig. 3). Beautifil demonstrated relatively stable L* values with a modest increase following repolishing in some groups and minor variations in a* values across polishing protocols, suggesting partial color recovery after surface cleaning. While b* values increased following brushing and showed a partial reduction after repolishing, these changes could be attributed to the hydrophilic Bis-GMA/TEGDMA matrix and variations in filler technology. The inclusion of S-PRG filler, which enhances fluoride release, might create vacancies on the composite surface, thereby promoting stainability [11]. Moreover, a study by Gonulol et al. reported that the increased water sorption of giomers reduces surface stain resistance compared with nanohybrid composites [13, 46] (Figs. 5, 6 and 7).

Another possible explanation is that the tested nanofilled composite is a paste-like, packable material that is more likely to trap air bubbles during application and sculpting. In contrast, both injectable composites, Beautifil Flow Plus X F00 and G-ænial Injectable, have a flowable consistency and were injected directly into the mold using specially designed plungers without sculpting, thereby reducing the likelihood of air bubble entrapment. However, the presence of porosity in putty composite materials increases their water absorption, which can lead to the accumulation of stains [41]. The varied results, supported by differences in consistency among the tested materials, align with the findings of Bai et al. [15]. In both studies, G-aenial Universal Injectable exhibited favorable optical behavior, which may be attributed to its superior performance in water sorption and solubility compared to other tested materials. However, there was no staining challenge in Bai et al. ‘s study, and the Filtek materials differed. Bai et al. reported favorable color stability for Filtek Supreme Flowable, while the present study showed greater stain susceptibility for Filtek Z350 XT in the putty form. This supports that differences in material viscosity, resin matrix composition, filler characteristics, and surface behavior between highly filled flowable and putty formulations affect water sorption, stain adsorption, and color recovery [47].

Moreover, the unpolished control specimens (Mylar strip) exhibited the highest ΔE_00_ values, confirming that the superficial resin-rich layer is more susceptible to pigment absorption. This finding aligns with previous studies by Kumari et al. and Jardy et al., who emphasized the importance of removing the outer layer to enhance resistance to staining [8, 44]. The Mylar strip, as evidenced in the literature, produces the smoothest surface. However, it is less color-stable due to oxidation. Nevertheless, essential clinical steps such as excess material removal, restoration, and recontouring can alter the results obtained with the strip matrix [8, 26]. Clinically, this emphasizes the necessity of finishing and polishing to eliminate this layer, thereby ensuring the aesthetic success and longevity of composite restoration.

Researchers have reported that no single polishing system performs equally well across all composite resins and that multi-step systems outperform single-step systems [42]. Our findings corroborate previous research, suggesting that a multi-step polishing protocol is more effective at achieving a stain-resistant surface, regardless of the resin type [48]. The abrasive particles used in polishing systems should not only be harder than the composites’ fillers but also smaller in size to prevent scratches on the composite surface [49, 50]. Consistent with most of the literature, multi-step diamond-based polishing systems, especially Enamel Plus Shiny, offer superior performance by gradually refining the composite surface. They abrade both filler and resin matrix phases uniformly, thereby producing smoother, glossier surfaces that resist discoloration [18, 37] (Fig. 4). Moreover, the ability of the polishing paste to move freely over the surface results in uniform wear of the restoration surface [8]. These findings align with our results, in which specimens polished with diamond pastes followed by aluminum oxide paste exhibited the least susceptibility to staining and the best color recovery among all composite types. In contrast, one-step aluminum-based polishers, such as OneGloss, were associated with microcracks and surface defects that serve as niches for stain accumulation, due to their larger abrasive particle size (85 μm) compared to the filler sizes of the tested materials [16] **(**Tables 1 and 2**)**. This explains the significantly pronounced discoloration observed with the simplified one-step polishing protocol (OneGloss) (*p* < 0.05) (Table 6; Fig. 3). Ergücü et al. and Turkun & Turkun previously reported similar outcomes, showing that one-step systems compromise final surface quality [51]. Therefore, polishing simplification doesn’t guarantee surface stain resistance or possible color recovery.

### Clinical implications

1. Among all tested materials, only multi-step-polished G-aenial Injectable exhibited color change values within an acceptable range after repolishing, highlighting its superior polishability and improved surface stain resistance.
2. A multi-step polishing system not only reduces stain susceptibility but also improves color recovery upon professional repolishing with AL_2_O_3_ paste, whereas one-step polishers may require more aggressive repolishing techniques to achieve similar results.
3. Toothbrushing cannot substitute for professional repolishing in the removal of surface stains.
4. Professional repolishing procedures can provide a conservative and effective approach for improving color recovery, enhancing restoration longevity, and reducing the need for premature restoration replacement.
5. The selection of both the restorative material and the polishing protocol may influence the long-term esthetic performance of resin composite restorations.

### Limitations and future directions

Nonetheless, certain limitations should be acknowledged. As a laboratory study, the absence of saliva, enzymatic activity, and dynamic changes in oral pH limits the direct extrapolation of these findings to clinical practice. Continuous immersion in coffee may exaggerate discoloration compared to intermittent exposure in the oral environment. Clinically, thermal cycling and mechanical abrasion from diet and oral hygiene were not simulated, and repolishing procedures using the full polishing protocols were not incorporated, despite their known role in clinical stain management. The absence of surface roughness measurements, gloss correlation, and assessments of dimensional stability and changes in mechanical properties also prevented a comprehensive understanding of alterations in surface and intrinsic properties that directly affect stain susceptibility and color recovery. Eventually, only selected materials were investigated; therefore, findings cannot be generalized to all restorative systems. 

### Within the limitations of this study, the following conclusions can be drawn

1. G-aenial Injectable has lower staining susceptibility than Beautifil Flow Plus X and better color recovery upon repolishing.
2. Multi-step polishing improves stain resistance and color recovery compared to one-step systems.
3. The stain susceptibility appeared to be influenced by resin matrix characteristics, filler technology, and the surface polishability of the tested restorative materials.
4. Color recovery can be partially obtained by cleaning and repolishing.

## Supplementary Information

Supplementary Material 1.

## Data Availability

No datasets were generated or analysed during the current study.
