# Supplementary material for: Stain susceptibility of a giomer and a particulate-filled injectable composite with one-step and multi-step polishing techniques, and color recovery after brushing and repolishing
Source: BMC Oral Health. 2026 Aug 25;26:1594. doi: 10.1186/s12903-026-09651-2 (PMC13504834; doi:10.1186/s12903-026-09651-2)
Supplement: Supplementary file 1 [file 12903_2026_9651_MOESM1_ESM.docx]

**Table 1 CIE L* a*b* (Mean ± SD) of Filtek supreme Z350 SD**

|  |  | L* (Lightness) | a*(Red-Green) | b* (Yellow-Blue) |
| --- | --- | --- | --- | --- |
| POLISHING PROTOCOL | Measurement time | (Mean ± SD) | (Mean ± SD) | (Mean ± SD) |
| Myler strip | baseline (T0) | 72.24 ± 5.71 | 1.01 ± 0.48 | 20.13 ± 1.95 |
|  | Brushing (T1) | 65.59 ± 6.22 | 1.5 ± 2.23 | 36.41 ± 5.62 |
|  | Repolishing (T2) | 69 ± 5.58 | 0.87 ± 0.86 | 31.63 ± 4.81 |
| One step | baseline (T0) | 74.76 ± 5.18 | 1.17 ± 0.74 | 22.35 ± 1.42 |
|  | Brushing (T1) | 72.36 ± 2.22 | 0.81 ± 0.28 | 31.87 ± 1.6 |
|  | Repolishing (T2) | 74.37 ± 2.91 | 0.59 ± 0.24 | 29.39 ± 1.42 |
| Multi-steps | baseline (T0) | 69.44 ± 3.84 | 2.11 ± 0.78 | 21.29 ± 0.94 |
|  | Brushing (T1) | 72.7 ± 3.25 | 1.07 ± 0.73 | 24.8 ± 1.73 |
|  | Repolishing (T2) | 75.61 ± 3.05 | 0.7 ± 0.42 | 24.12 ± 1.09 |

**Table 2 CIE L* a*b* (Mean ± SD) of** **G-ænial Injectable**

|  |  | L* (Lightness) | a*(Red-Green) | b* (Yellow-Blue) |
| --- | --- | --- | --- | --- |
| POLISHING PROTOCOL | Measurement time | (Mean ± SD) | (Mean ± SD) | (Mean ± SD) |
| Myler strip | baseline (T0) | 63.25 ± 3.88 | 1.19 ± 0.68 | 17.47 ± 1.84 |
|  | Brushing (T1) | 68.29 ± 3.76 | 0.89 ± 0.54 | 23.23 ± 3.01 |
|  | Repolishing (T2) | 68.40 ± 3.44 | 1.06 ± 0.48 | 20.29 ± 2.62 |
| One step | baseline (T0) | 62.62 ± 5.08 | 0.72 ± 0.69 | 16.50 ± 1.71 |
|  | Brushing (T1) | 67.14 ± 5.05 | 1.51 ± 0.47 | 19.68 ± 1.56 |
|  | Repolishing (T2) | 68.00 ± 4.85 | 1.10 ± 0.66 | 17.79 ± 1.54 |
| Multi-steps | baseline (T0) | 66.04 ± 2.37 | 1.73 ± 0.63 | 18.47± 0.86 |
|  | Brushing (T1) | 67.57 ± 2.59 | 2.39 ± 0.58 | 20.96 ± 0.80 |
|  | Repolishing (T2) | 67.50± 1.69 | 2.42 ± 8.55 | 18.80 ± 0.76 |

|  |  | L* (Lightness) | a*(Red-Green) | b* (Yellow-Blue) |
| --- | --- | --- | --- | --- |
| POLISHING PROTOCOL | Measurement time | (Mean ± SD) | (Mean ± SD) | (Mean ± SD) |
| Myler strip | baseline (T0) | 66.01 ± 5.12 | 1.18 ± 0.51 | 16.83 ± 2.12 |
|  | Brushing (T1) | 70.53 ± 3.69 | 1.17 ± 0.91 | 23.09 ± 7.52 |
|  | Repolishing (T2) | 72.11 ± 4.6 | 0.8 ± 0.55 | 20.92 ± 6.16 |
| One step | baseline (T0) | 74.81 ± 5.64 | 1.42 ± 1.01 | 18.89 ± 1.27 |
|  | Brushing (T1) | 73.43 ± 4.03 | 1.19 ± 0.56 | 28.31 ± 0.99 |
|  | Repolishing (T2) | 76.35 ± 4.46 | 1.2 ± 0.8 | 23.54 ± 1.98 |
| Multi-steps | baseline (T0) | 70.02 ± 4.68 | 2.21 ± 0.95 | 19.23 ± 1.42 |
|  | Brushing (T1) | 66.07 ± 2.69 | 2.18 ± 0.82 | 22.8 ± 2.51 |
|  | Repolishing (T2) | 66.17 ± 2.08 | 2.42 ± 0.64 | 22.8 ± 2.51 |

**Table 3 CIE L* a*b* (Mean ± SD) of Beautifil F00 (Giomer)**
